# Coronavirus-19: Possible Therapeutic Implications of Spironolactone and Dry Extract of *Glycyrrhiza glabra* L. (Licorice)

**DOI:** 10.3389/fphar.2020.558418

**Published:** 2020-10-22

**Authors:** Decio Armanini, Cristina Fiore, Jens Bielenberg, Chiara Sabbadin, Luciana Bordin

**Affiliations:** ^1^ Department of Medicine-Endocrinology, University of Padua, Padua, Italy; ^2^ Department of Molecular Medicine-Biological Chemistry, University of Padua, Padua, Italy

**Keywords:** spironolactone, licorice, androgen receptor, inflammation, plasma membrane

## Abstract

https://clinicaltrials.gov/ (NCT044241349, NCT043465887, NCT04487964)

The membrane angiotensin converting enzyme 2 (ACE2) couples to the spike protein of Coronavirus-19 (Covid-19), inducing proteolytic cleavage by a cathepsin transmembrane protease serine 2 (TMPRSS2), and fusion of the viral and cellular membranes (Hoffman et al., 2020). ACE2 is also present in a circulating soluble form that lacks the membrane anchor (Batlle et al., 2020). The virus seems to infect not only epithelial cells in the lung, but also inflammatory cells, inducing local secretion of cytokines. Co-expression of ACE2 and TMPRSS2 has frequently been found in the upper and lower respiratory tract in SARS-coronavirus target cells and could thus support viral spread in the human host and between individuals (Bertram et al., 2012).

The positive or negative implications of treatment with ACE inhibitors and angiotensin II receptor blockers (ARB) in infected hypertensive patients is the object of studies and interpretations. The spike protein of the SARS-CoV reduces ACE2 expression and worsens acute lung failure *in vivo*. This effect can be attenuated in experimental studies by blocking the renin-angiotensin pathway (Imai et al., 2005). A study on ACE2 knockout mice provides a molecular explanation of why SARS-CoV infections cause severe and often lethal lung failure and suggests a rational therapy with recombinant ACE or with drugs that increase angiotensin II and ACE2 (Imai et al., 2005; Murthy et al., 2020). A possible explanation of the usefulness of therapy with drugs that increase circulating ACE 2 is that the binding of the virus to circulating ACE reduces its binding to ACE2 receptor of the cells of the tissues that possess the TMPRSS2.

In this short excursus we aim to highlight a possible positive effect on Covid-19 infections of spironolactone (SP) and of glycyrrhizin (GI), the main component of *Glycyrrhiza glabra* L. (licorice) root. SP increases the circulating ACE2 (Keidar et al., 2005; Kong et al., 2019; Stoll et al., 2019) and also has a powerful anti-inflammatory effect, antagonizing the inflammatory and cardiovascular effects of aldosterone even when its concentration is normal (Armanini et al., 2014). A recent study has shown that patients treated with antiandrogens for prostate cancer are protected from the coronavirus infection. This effect could be related to the blocking of TMPRSS2 (Stopsach et al., 2020). SP not only combines antialdosterone and antiandrogen activity by antagonizing both receptors, but also has a direct action in the adrenals, ovary and testes, where it locally blocks some enzymes involved in the synthesis of aldosterone and androgens. SP also has a stimulatory effect on aromatase (Armanini et al., 2014). Both these properties of SP could have a role in patients with Covid-19 infection.

A possible additive treatment with licorice could be considered, based on a report of the antiviral effect of licorice in SARS-coronavirus and in other virus diseases. In 2003, during the SARS-coronavirus epidemic, Cinatl and collaborators reported that GI, the main active principle of licorice, inhibits SARS-related coronavirus replication in VERO cells *in vitro*. Unlike ribavirin, GI was able to reduce the absorption of the virus during the early steps of replication. A possible explanation is that GI induces nitrous oxide synthase, as virus replication was inhibited by addition of this compound (Cinatl et al., 2003).

Licorice is one of the most used medicinal plants since antiquity. Numerous ancient reports mention its use for symptoms attributable to respiratory tract infections, such as dry cough and hoarseness (Yeh et al., 2013), and recent studies have reported an antiviral effect of licorice (Fiore et al., 2008b; Hardy et al., 2012; Wang et al., 2015). GI decreases liver enzymes and improves the histological picture of chronic hepatitis C. In mice infected with Influenza virus A2, pulmonary consolidation and viral load in the lungs were significantly lower following treatment with GI than with saline. In the same model the splenic T cells of GI-treated mice were transferred to mice infected with the virus and all recipients survived. The authors attributed the effect to the stimulation of interferon secretion by immunocompetent cells (Utsunomiya et al., 1997). GI can inhibit HIV replication in mononuclear cell cultures of infected patients (Sasaki et al., 2002–2003). GI also has a protective effect on the plasma membrane of cells, which could be involved in its antiviral activity. A direct effect of GI on the cell membrane has been reported in human erythrocytes, where GI, in a dose-dependent manner, strengthens membrane integrity against both oxidative and proteolytic damage of band 3 membrane protein, induced *in vitro* (Fiore et al., 2008).

A similar protective effect is exerted by canrenone, the main metabolite of SP, in erythrocytes of patients with primary aldosteronism (Bordin et al., 2013). Based on these reports, the antiviral effect of GI seems independent of an effect on ACE2 in lung endothelial cells. GI could be evaluated for a possible enhancement of immune defences against virus infection and for protection of the plasma membrane from virus penetration. The main problem for the clinical use of licorice, however, is its mineralocorticoid-like effect, due to the block type 2 11‐hydroxysteroid dehydrogenase and to binding of GI to mineralocorticoid receptors (Sabbadin et al., 2019). Licorice can be associated with SP and this association has already been reported in the treatment of women with polycystic ovary syndrome, where pure licorice extract (at the dose of 3.5 g a day) enhances the antiandrogen and anti-inflammatory and thrombolytic effect of SP, hereby blocking the risk of hyperkalemia and hypotension (Sabbadin et al., 2019).

The mechanisms of action of SP and licorice are summarized in the Table 1. While this manuscript was reviewed, other papers were published (Cadegiani, 2020; Chen et al., 2020; Liaudet and Szabo, 2020; Luo et al., 2020; Murck, 2020; Zhao et al., 2020). There are some ongoing trials with SP in patients with COVID-19 disease, as reported in clinicaltrials.gov website. One is focused on changes from baseline in clinical assessment score and laboratory parameters during treatment with SP and bromexine (NCT044241349). The other is related to SP induced improvement in acute respiratory distress syndrome and in sequential organ failure assessment (NCT043465887).

**TABLE 1:** SP and licorice: comparative effects.

Mechanisms of the action of SP
Increased circulating ACE2, limiting the access of the virus to cell membranes
Powerful anti-androgen and anti-mineralocorticoid effect with decreased expression of TMPRSS2 and decreased production of inflammatory cytokines
Block of the membrane damage caused by MR activation in inflammatory situations
Block of the hypertensive and hypokalemic effects of licorice
Effects of licorice actions
Preservation of membrane integrity
Stimulation of interferon gamma secretion
Anti-inflammatory effect
Partial block of TMPRSS2 due to the reduction of androgen secretion by testis, ovary and adrenals
Control of the hypotensive and hyperkalemic effect of SP

Regarding to licorice there is one on-going pilot, non randomized study with licorice extract as a complementary medicine in patients with COVID-19 disease (NCT04487964, but nobody has proposed the association licorice and SP.

In conclusion, licorice does not need prescription and is available in many countries as a pure extract. We propose that the possible association of GI and SP may be considered for Covid-19 infections, taking account of the different mechanism of action of the two substances and their synergism. The additive activity of SP and licorice combination should be presented as a potential mechanism, not currently supported by evidence derived from clinical trials. Moreover, it should be mentioned that, as an extract, licorice would require a careful quantitative assessment of its active principle GI to define dose-response relationship.

## Author Contributions

DA, CF, JB, CS and LB conceived of the hypothesis and contributed to the draft and revision of the manuscript.

## Conflict of Interest

The authors declare that the research was conducted in the absence of any commercial or financial relationships that could be construed as a potential conflict of interest.

## References

[B1] Armanini D. Sabbadin C. Donà G. Clari G. Bordin L. (2014). Aldosterone receptor blockers spironolactone and canrenone: two multivalent drugs. Expet Opin. Pharmacother. 15 (7), 909–912. 10.1517/14656566.2014.896901 10.1517/14656566.2014.896901 | Google Scholar 24617854

[B2] Batlle D. Wysocki J. Satchell K. (2020). Soluble angiotensin-converting enzyme 2: a potential approach for coronavirus infection therapy?. Clin. Sci. (Lond.) 134 (5), 543–545. 10.1042/CS20200163 10.1042/CS20200163 | Google Scholar 32167153

[B3] Bertram S. Heurich A. Lavender H. Gierer S. Danisch S. Perin P. (2012). Influenza and SARS-coronavirus activating proteases TMPRSS2 and HAT are expressed at multiple sites in human respiratory and gastrointestinal tracts. PLoS One 7 (4), e35876 10.1371/journal.pone.0035876 10.1371/journal.pone.0035876 | Google Scholar 22558251 PMC3340400

[B4] Bordin L. Donà G. Ragazzi E. Andrisani A. Ambrosini G. Brunati A. M. (2013). Human red blood cells alterations in primary aldosteronism. J. Clin. Endocrinol. Metab. 98 (6), 2494–2501. 10.1210/jc.2012-3571 10.1210/jc.2012-3571 | Google Scholar 23539731

[B5] Cadegiani F. A. (2020). Can spironolactone be used to prevent COVID-19-induced acute respiratory distress syndrome in patients with hypertension?. Am. J. Physiol. Endocrinol. Metab. 318 (5), E587–E588. 10.1152/ajpendo.00136.2020 10.1152/ajpendo.00136.2020 | Google Scholar 32297520 PMC7191632

[B6] Chen L. Hu C. Hood M. Zhang X. Zhang L. Kan J. (2020). A novel combination of vitamin C, curcumin and glycyrrhizic acid potentially regulates immune and inflammatory response associated with coronavirus infections: A perspective from system biology analysis. Nutrients 12, 1193 10.3390/nu12041193 10.3390/nu12041193 | Google Scholar PMC7230237 32344708

[B7] Cinatl J. Morgenstern B. Bauer G. Chandra P. Rabenau H. Doerr H. W. (2003). Glycyrrhizin, an active component of liquorice roots, and replication of SARS-associated coronavirus. Lancet 361 (9374), 2045–2046. 10.1016/s0140-6736(03)13615-x 10.1016/s0140-6736(03)13615-x | Google Scholar 12814717 PMC7112442

[B8] Fiore C. Bordin L. Plellati D. Armanini D. Clari G. (2008a). Effect of glycyrrhetinic acid on membrane band 3 in human erythrocytes. Arch. Biochem. Biophys. 479, 46–51. 10.1016/j.abb.2008.08.011 10.1016/j.abb.2008.08.011 | Google Scholar 18778682

[B9] Fiore C. Eisenhut M. Ragazzi E. Pellati D. Armanini D. Bielenberg J. (2008b). Antiviral Effects of Glycyrrhiza species. Phytother. Res. 22, 141–148. 10.1002/ptr.22959 10.1002/ptr.22959 | Google Scholar 17886224 PMC7167979

[B10] Hardy M. E. Hendricks J. M. Paulson J. M. Faunce N. R. (2012). 18β-glycyrrhetinic acid inhibits rotavirus replication in culture. Virol. J. 9, 96 10.1186/1743-422X-9-96 10.1186/1743-422X-9-96 | Google Scholar 22616823 PMC3478227

[B11] Hoffmann M. Kleine-Weber H. Schroeder S. Krüger N. Herrler T. Erichsen S. (2020). SARS-CoV-2 cell entry depends on ACE2 and TMPRSS2 and is blocked by a clinically proven protease inhibitor. Cell Open 181 (2), 271–280. 10.1016/j.cell.2020.02.052 10.1016/j.cell.2020.02.052 | Google Scholar PMC7102627 32142651

[B12] Imai Y. Kuba K. Rao S. Huan T. Guo F. Guan B. (2005). Angiotensin-converting enzyme 2 protects from severe acute lung failure. Nature 436 (7047), 112–116. 10.1038/nature03712 10.1038/nature03712 | Google Scholar 16001071 PMC7094998

[B13] Keidar S. Gamliel-Lazarovich A. Kaplan M. Pavlotzky E. Hamoud S. Hayek T. (2005). Mineralocorticoid receptor blocker increases angiotensin-converting enzyme 2 activity in congestive heart failure patients. Circ. Res. 97 (9), 946–953. 10.1161/CIRCRESAHA.120.317174 10.1161/CIRCRESAHA.120.317174 | Google Scholar 16179584

[B14] Ko H. C. Wei B. L. Chiou W. F. (2006) The effect of medicinal plants used in Chinese folk medicine on RANTES secretion by virus-infected human epithelial cells. J. Ethnopharmacol. 107 (2), 205–210. 10.1016/j.jep.2006.03.004 10.1016/j.jep.2006.03.004 | Google Scholar 16621378

[B15] Kong E. Zhang J. An N. Tao Y. Yu W. Wu F. (2019). Spironolactone rescues renal dysfunction in obstructive jaundice rats by upregulating ACE2 expression. J. Cell Commun. Signal. 13 (1), 17–26. 10.1007/s12079-018-0466-2 10.1007/s12079-018-0466-2 | Google Scholar 29882088 PMC6381374

[B16] Liaudet L. Szabo C. (2020). Blocking mineralocorticoid receptor with spironolactone may have a wide range of therapeutic actions in severe COVID-19 disease. Crit. Care 24 (1), 318 10.1186/s13054-020-03055-6 10.1186/s13054-020-03055-6 | Google Scholar 32513242 PMC7278213

[B17] Luo P. Liu D. Li J. (2020). Pharmacological perspective: glycyrrhizin may be an efficacious therapeutic agent for COVID-19. Int. J. Antimicrob. Agents 55, 105995 10.1016/j.ijantimicag.2020.105995 CrossRef Full Text | Google Scholar 32335281 PMC7180159

[B18] Murck H. (2020). Symptomatic protective action of Glycyrrhizin (Licorice) in COVID-19 infection? Front. Immunol. 11, 1239 10.3389/fimmu.2020.01239 10.3389/fimmu.2020.01239 | Google Scholar 32574273 PMC7270278

[B19] Murthy V. L. Koupenova M. Shah R. V. (2020). ACEing COVID-19: a role for angiotensin axis inhibition in SARS-CoV-2 infection?. Circ. Res. 126 (12), 1682–1684. 10.1161/CIRCRESAHA.120.317174 10.1161/CIRCRESAHA.120.317174 | Google Scholar 32302248 PMC7274880

[B20] Sabbadin C. Bordin L. Donà G. Manso J. Avruscio G. Armanini D. (2019). Licorice: from pseudohyperaldosteronism to therapeutic uses. Front. Endocrinol. 10, 484 10.3389/fendo.2019.00484 10.3389/fendo.2019.00484 | Google Scholar PMC6657287 31379750

[B21] Sasaki H. Takei M. Kobayashi M. Pollard R. B. Suzuki F. (2002–2003). Effect of glycyrrhizin, an active component of licorice roots, on HIV replication in cultures of peripheral blood mononuclear cells from HIV-seropositive patients. Pathobiology 70 (4), 229–236. 10.1159/000069334 10.1159/000069334 | Google Scholar 12679601

[B22] Stoll R. Yokota R. Sanches Aragã D. Casarini D. E. (2019). Both aldosterone and spironolactone can modulate the intracellular ACE/ANG II/AT1 and ACE2/ANG (1-7)/MAS receptor axes in human mesangial cells. Physiol. Rep. 7 (11), e14105 10.14814/phy2.14105 10.14814/phy2.14105 | Google Scholar 31165585 PMC6548847

[B23] Stopsack K. H. Mucci L. A. Antonarakis E. Nelson P. S. Kantoff P. W. (2020). TMPRSS2 and COVID-19: serendipity or opportunity for intervention?. Cancer Discov. 10, 779–782. 10.1158/2159-8290.CD-20-0451 10.1158/2159-8290.CD-20-0451 | Google Scholar 32276929 PMC7437472

[B24] Utsunomiya T. Kobayashi M. Pollard R. B. Suzuki F. (1997). Glycyrrhizin, an active component of licorice roots, reduces morbidity and mortality of mice infected with lethal doses of influenza virus. Antimicrob. Agents Chemother. 41 (3), 551–556. 10.1128/AAC.41.3.551 10.1128/AAC.41.3.551 | Google Scholar 9055991 PMC163749

[B25] Wang L. Yang R. Yuan B. Liu Y. Liu C. (2015). The antiviral and antimicrobial activities of licorice, a widely-used Chinese herb. Acta Pharm. Sin. B 5 (4), 310–315. 10.1016/j.apsb.2015.05.005 10.1016/j.apsb.2015.05.005 | Google Scholar 26579460 PMC4629407

[B26] Yeh C. F. Wang K. C. Chiang L. C. Shieh D. E. Yen M. H. Chang J. S. (2013). Water extract of licorice had anti-viral activity against human respiratory syncytial virus in human respiratory tract cell lines. J. Ethnopharmacol. 148, 466–473. 10.1016/j.jep.2013.04.040 10.1016/j.jep.2013.04.040 | Google Scholar 23643542 PMC7126896

[B27] Zhao X. Jiang Y. Zhao Y. Xi H. Liu C. Qu F. (2020). Analysis of the susceptibility to COVID-19 in pregnancy and recommendations on potential drug screening. Eur. J. Clin. Microbiol. Infect. Dis. 39 (7), 1209–1200. 10.1007/s10096-020-03897-6 10.1007/s10096-020-03897-6 | Google Scholar 32328850 PMC7178925

